# LncRNA Neat1 targets NonO and miR-128-3p to promote antigen-specific Th17 cell responses and autoimmune inflammation

**DOI:** 10.1038/s41419-023-06132-0

**Published:** 2023-09-16

**Authors:** Sisi Chen, Jiali Wang, Kailang Zhang, Binyun Ma, Xiaorong Li, Ruihua Wei, Hong Nian

**Affiliations:** 1 Tianjin Key Laboratory of Retinal Functions and Diseases, Tianjin Branch of National Clinical Research Center for Ocular Disease, Eye Institute and School of Optometry, Tianjin Medical University Eye Hospital, Tianjin, 300384 China; 2 Department of Medicine/Hematology, Keck School of Medicine of the University of Southern California, Los Angeles, CA 90033 USA

**Keywords:** Immunological disorders, T cells, Immunological disorders

## Abstract

Long non-coding RNAs (lncRNAs) interaction with RNA-Binding proteins (RBPs) plays an important role in immunological processes. The generation of antigen-specific Th17 cells is closely associated with autoimmune pathogenesis. However, the function of lncRNA-RBP interactions in the regulation of pathogenic Th17 cell responses during autoimmunity remains poorly understood. Here, we found that lncRNA Neat1, highly expressed in Th17 cells, promoted antigen-specific Th17 cell responses. Both global and CD4^+^ T cell-specific knockdown of Neat1 protected mice against the development of experimental autoimmune uveitis (EAU). Mechanistically, Neat1 regulated RNA-Binding protein NonO, thus relieving IL-17 and IL-23R from NonO-mediated transcriptional repression and supporting antigen-specific Th17 cell responses. In addition, Neat1 also modulated miR-128-3p/NFAT5 axis to increase the expression of IL-17 and IL-23R, leading to augmented Th17 cell responses. Our findings elucidate a previously unrecognized mechanistic insight into the action of Neat1 in promoting antigen-specific Th17 responses and autoimmunity, and may facilitate the development of therapeutic targets for T cell-mediated autoimmune diseases.

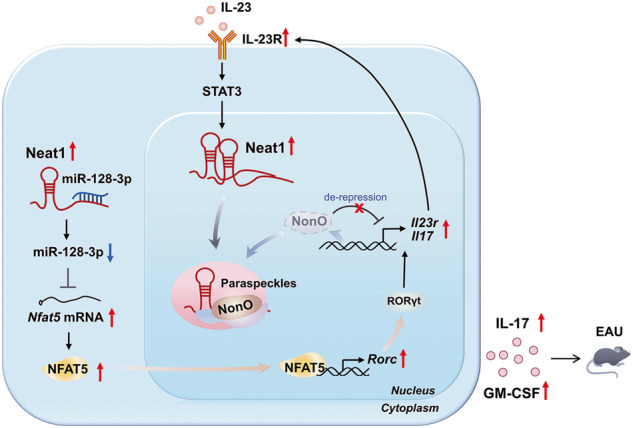

## Introduction

Autoimmune uveitis (AU) is an immune-mediated intraocular inflammatory disorder characterized by inflammatory cell infiltration and ocular damage, which eventually leads to visual impairment and blindness [1]. AU affects millions of people worldwide and occurs mainly in young adults, with significant personal and socioeconomic burdens [2]. The etiology of AU is complex and ambiguous, involving genetic, pathological, and environmental factors [3]. Experimental autoimmune uveitis (EAU), an established mouse model of human AU, has been instrumental in the development of AU therapies [4]. Antigen-specific Th17 cells are key players in the pathogenesis of many autoimmune disorders including AU and EAU [5, 6]. IL-23/IL-23R signaling has been considered pivotal for pathogenicity in Th17 cells [7–9]. Clinically, the level of IL-23 in the serum obtained from patients with active AU was significantly higher than that of patients with inactive AU and healthy controls [8]. In addition, the enhanced expression of IL-17 and IL-23R was observed in the peripheral blood mononuclear cells (PBMCs) of patients with active AU [9, 10]. Moreover, pre-clinical animal studies showed that the adoptive transfer of activated interphotoreceptor retinoid-binding protein (IRBP)-specific Th17 cells into naïve mice could induce EAU, while suppression of antigen-specific Th17 cell responses could alleviate EAU [11, 12], suggesting the importance of controlling pathogenic Th17 cells and its regulatory pathways in AU treatment. Currently, several emerging drugs blocking IL-17 or Th17 cell regulatory pathways, such as Ustekinumab, Adalimumab, and Secukinumab et al., have been shown effective in the treatment of AU in pilot clinical studies [9]. Therefore, efforts focused on finding novel molecular mechanisms underlying the regulation of pathogenic Th17 cell responses will have a potential impact on AU treatment.

Long noncoding RNAs (lncRNAs), a cluster of non-protein-encoding RNAs with longer than 200 nucleotides, play crucial roles in various physiological and pathological processes, including autoimmune disorders [13–15]. A recent study found that single nucleotide polymorphisms (SNPs) rs3829794 in lnc-TOR3A-1:1 conferred susceptibility to Vogt-Koyanagi-Harada disease-associated AU [16], suggesting that lncRNAs are closely related to the pathogenesis of AU. Nevertheless, the roles and mechanisms of lncRNAs in the development of AU remain in need of further investigation. LncRNA Nuclear paraspeckle assembly transcript 1 (Neat1), transcribed from the multiple endocrine neoplasia (Men) locus, is an important regulator of immune responses [17–20]. Neat1 can promote macrophage polarization to M1 phenotype via modulating miR-188-5p/BTK axis [18], and promote Th2 cell differentiation by inhibiting STAT6 ubiquitination [19]. However, whether and how Neat1 regulates antigen-specific Th17 cells and EAU remains unknown, although a study reported its role in the regulation of the differentiation of Th17 cells from naïve CD4^+^ T cells.

As a multifunctional DNA- and RNA-Binding protein, the non-POU domain containing octamer binding protein (NonO) has been recently shown to play a pivotal role in several biological processes via regulation of gene transcription or translation [21–23]. For example, NonO functions as an RBP to interact with c-Myc mRNAs and facilitate the internal ribosome entry segment (IRES)-dependent translation of c-Myc, thus reducing HeLa cell apoptosis [24]. It can also directly bind to the *Il6* promoter and act together with PRDM1 to inhibit dendritic cell activation [25]. Recent studies showed that lncRNAs interaction with RBPs plays critical roles in immunological processes [15, 26, 27]. Nevertheless, the function of lncRNA-RBP interactions in the regulation of pathogenic Th17 cell responses during autoimmunity remains poorly understood.

In this study, we examined the functional role of Neat1 in the regulation of antigen-specific Th17 cell responses in EAU. Our findings revealed that Neat1 promoted antigen-specific Th17 cells via regulating NonO and sponging miR-128-3p. These data offer important insight into pathogenic Th17 cell biology and may provide promising therapeutic targets for Th17-related autoimmune diseases.

## Results

### lncRNA Neat1 is upregulated in the PBMCs of AU patients and antigen-specific Th17 cells in EAU

**Fig. 1:**
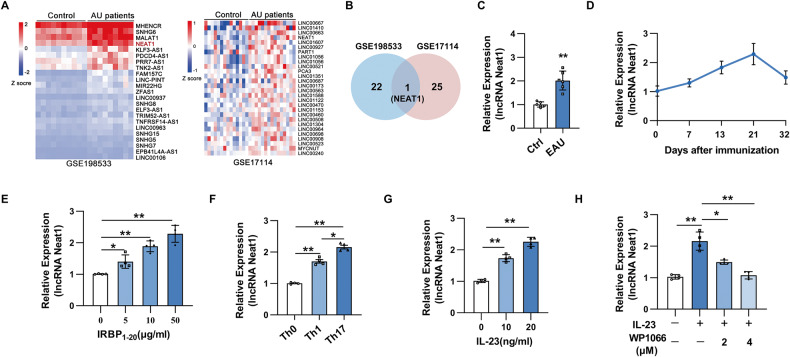
Neat1 is upregulated in the PBMCs of AU patients and antigen-specific Th17 cells of EAU mice. **A** Heatmap of upregulated lncRNAs in PBMCs of AU patients compared with healthy controls. Upregulated lncRNAs were sorted via analysis of dataset GSE198533 (including 10 BD patients and 10 healthy controls) and GSE17114 (containing 15 BD patients and 14 healthy controls). **B** Venn diagram showing the overlap between lncRNAs upregulated in two publicly available datasets of AU patients (GSE17114 and GSE198533). **C** Real-time qRT-PCR analysis of Neat1 expression in CD4^+^ T cells from naïve (Ctrl) and EAU mice (*n* = 6 per group). **D** Kinetic expression of Neat1 expression in CD4^+^ T cells during the course of EAU (*n* = 4 per group). **E**–**H** CD4^+^ T cells were isolated from EAU mice and co-cultured with APCs and IRBP_1-20_ under indicated conditions for 48 h. Real-time qRT-PCR analysis of Neat1 expression (*n* = 4 per group). Data are presented as mean ± SD of at least three independent experiments. *p < 0.05, **p < 0.01.

To identify lncRNAs involving in AU progression, we analyzed the publicly available database of RNA-seq (GSE198533) and RNA microarray (GSE17114) of PBMCs derived from AU patients. Among the upregulated lncRNAs, we found that only lncRNA NEAT1 was significantly upregulated in both datasets (Fig. 1A, B). To examine if Neat1 expression is associated with the development of EAU, we detected the expression of Neat1 in CD4^+^ T cells of EAU mice on day 13 after immunization. As shown in Fig. 1C, we observed that Neat1 was dramatically increased in CD4^+^ T cells of EAU mice compared with that of naïve mice. Further analysis revealed that the expression of Neat1 positively correlated with disease severity, with the highest level being observed at the disease peak (day 21 after immunization) (Fig. 1D). In addition, we found that a significant increase of Neat1 expression in EAU CD4^+^ T cells in response to increasing doses of IRBP_1–20_ (Fig. 1E). We further examined its expression in antigen-specific Th cell subsets and found that Neat1 was significantly increased in antigen-specific Th17 cells compared with that in Th0 and Th1 cells (Fig. 1F).

Given that Th17 cells require IL-23 signaling for their pathogenicity, we then explored whether IL-23 can regulate the expression of Neat1. As presented in Fig. 1G, IL-23 promoted the expression of Neat1 in a dose-dependent manner. STAT3 is one of the major downstream molecules mediating IL-23 signaling. To confirm the downstream signaling by which IL-23 induced the expression of Neat1, we pretreated CD4^+^ T cells with WP1066, a STAT3 inhibitor, before IL-23 stimulation. We found that treatment with WP1066 partially abolished IL-23-induced Neat1 expression in CD4^+^ T cells (Fig. 1H). Together, these results suggest that Neat1 induced by IL-23/STAT3 signaling may play an important role in the progression of EAU.

### Neat1-silenced mice developed alleviated EAU and displayed decreased Th17 cell responses

**Fig. 2:**
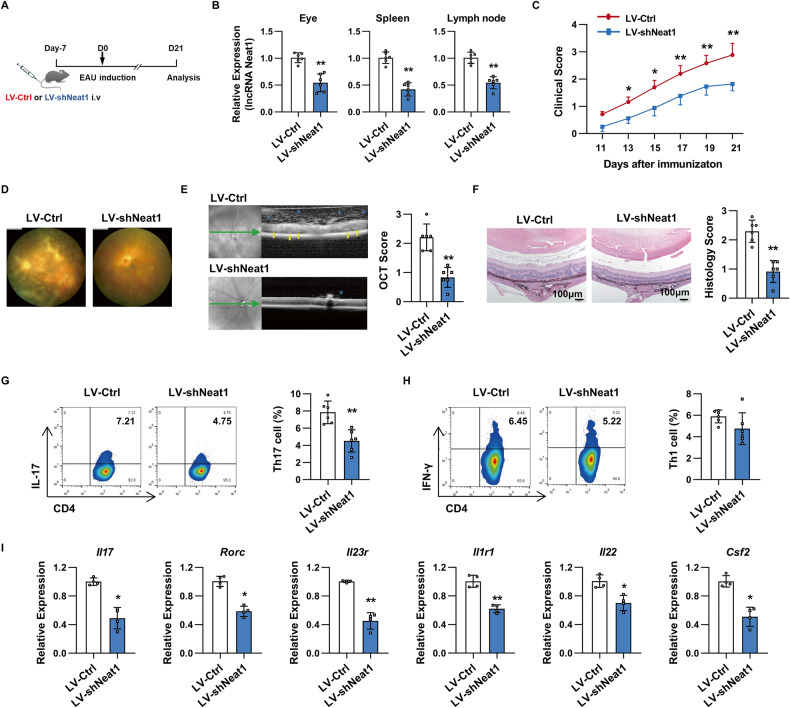
Neat1-silenced mice developed alleviated EAU and displayed decreased Th17 cell responses. **A** Schematic diagram of the experimental procedure to study the role of Neat1 in EAU. **B** Real-time qRT-PCR analysis of Neat1 expression in eyes, spleens, and lymph nodes of mice infected with LV-Ctrl or LV-shNeat1 (*n* = 6 per group). **C** Clinical score of EAU in mice infected with LV-Ctrl and LV-shNeat1 (*n* = 6 per group). **D** Representative images of eyes in LV-Ctrl and LV-shNeat1-infected EAU mice on day 21 after EAU induction by retinal imaging microscope. **E** Representative images showing fundus condition by SD-OCT. Hyper-reflective foci (blue asterisk) in the vitreous cavity and retinal folds (yellow arrows) were observed around the optical nerve. **F** Histopathological analysis of eyes from lentivirus-infected EAU mice (*n* = 6 per group). Scale bars, 100 μm. **G**, **H** Flow cytometric analysis of the percentages of Th17 (**G**) and Th1 (**H**) in T cells from lentivirus-infected EAU mice (*n* = 6 per group). **I** Real-time qRT-PCR analysis of pathogenic Th17-related genes in T cells from lentivirus-infected EAU mice (*n* = 4 per group). Data are presented as mean ± SD of at least three independent experiments. *p < 0.05, **p < 0.01. See also Fig. S1.

To determine the potential role of Neat1 in EAU pathogenesis, we delivered approximately 5 × 10^7^ transforming units of lentivirus to mice via tail vein injection to silence Neat1 expression before EAU induction (Fig. 2A). The efficiency of Neat1 knockdown in vivo was assessed by real-time qRT-PCR analysis. Neat1 levels were significantly reduced in the eyes, spleens, and lymph nodes of LV-shNeat1-infected mice (Fig. 2B). After immunization, we monitored the development of EAU via indirect ophthalmoscope. As shown in Fig. 2C, Neat1-silenced mice displayed remarkedly decreased EAU severity throughout the whole disease course. We observed that, compared to clinical scores of 2.88 ± 0.43 in control mice at day 21 post immunization, those in Neat1-silenced mice were significantly lower, with an average score of 1.82 ± 0.25 (Fig. 2C). To observe EAU more objectively, retinal imaging and SD-OCT analysis were performed on day 21 after EAU induction. The representative fundus images showed that more severe vasculitis, multiple chorioretinal lesions, and prominent inflammatory infiltration were observed in the fundus of control mice than that in Neat1-silenced mice (Fig. 2D). SD-OCT imaging, a non-invasive approach for visualizing the microstructure of fundus, revealed that knockdown of Neat1 significantly decreased inflammatory infiltrates in the vitreous cavity and optic nerve head compared with the control mice (Fig. 2E). Additionally, minimal retinal folds and photoreceptor inner-outer segment (IS/OS) junction disruptions were seen in the Neat1-silenced mice compared to control mice (Fig. 2E). Consistently, histopathological examination of eyes on day 21 after EAU induction showed that inflammatory infiltration and retinal damage were dramatically reduced in Neat1-silenced mice compared with the control mice (0.92 ± 0.38 vs. 2.30 ± 0.38, Fig. 2F).

CD4^+^ T cell subsets including Th1 and Th17 cells are closely involved in the pathogenesis of EAU. To examine the cellular mechanisms by which silencing Neat1 alleviated EAU, T cells from the spleens and lymph nodes from Neat1-silenced and control mice were analyzed by flow cytometry and real-time qRT-PCR. Compared with the control mice, the percentage of Th17 cells was dramatically decreased in the Neat1-silenced mice (Fig. 2G), while the frequency of Th1 cells had no significant difference between the two groups (Fig. 2H). In line with the changes in Th17 cell frequencies, silencing Neat1 resulted in a significantly decreased expression of pathogenic Th17-signature genes, such as *Il17*, *Rorc*, *Il23r*, *Il1r1*, *Il22*, and *Csf2* (Fig. 2I). In addition, we also observed that a significant increase in the proportion of Treg cells in the Neat1-silenced mice (Fig. S1A), and the expression of Treg-related genes, including *Il10*, *Foxp3*, and *Ahr*, was markedly augmented in T cells from Neat1-silenced mice (Fig. S1B).

Taken together, these data indicate that Neat1 promotes EAU mainly by affecting the Th17 cell responses.

### Silencing of Neat1 impairs antigen-specific Th17 cell responses in vitro

**Fig. 3:**
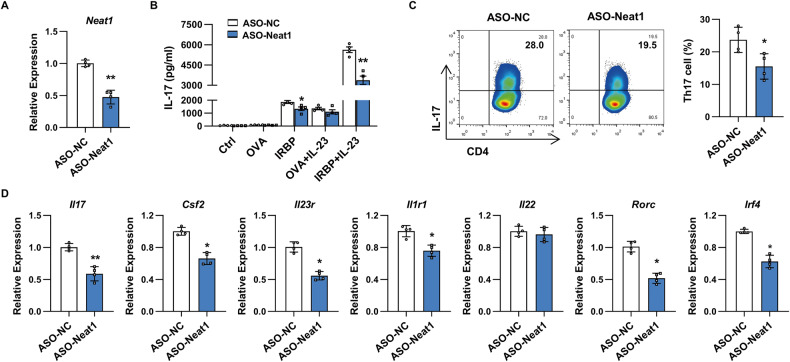
Neat1 downregulated in Th17 cells inhibits antigen-specific Th17 cell responses in vitro. **A**, **C**, **D** CD4^+^ T cells isolated from immunized mice were transfected with ASO-NC or ASO-Neat1 and stimulated with IRBP_1-20_ in the presence of irradiated APCs under Th17-polarizing conditions. **A** Real-time qRT-PCR analysis of Neat1 expression (*n* = 4 per group). **C** Flow cytometric analysis of the percentages of Th17 cells (*n* = 4 per group). **D** Real-time qRT-PCR analysis of Th17-related gene expression (*n* = 4 per group). **B** ELISA analysis of IL-17 production in CD4^+^ T cells transfected with ASO-NC or ASO-Neat1 and restimulated with the indicated antigens and IL-23 (*n* = 4 per group). Data are presented as mean ± SD of at least three independent experiments. *p < 0.05, **p < 0.01. See also Fig. S2.

In light of Neat1 promoting Th17 cell responses in vivo, we next asked whether Neat1 affected antigen-specific Th17 responses. To this end, CD4^+^ T cells isolated from EAU mice were transfected with antisense oligonucleotides targeting Neat1 (ASO-Neat1) or ASO-NC, washed, and then restimulated with immunized antigen (IRBP_1-20_) or irrelevant antigen (OVA_323-339_) in the presence of antigen-presenting cells (APCs) under Th17-polarizing conditions. As presented in Fig. 3A, B, knockdown of Neat1 led to significantly reduced IL-17 secretion when T cells were restimulated with IRBP_1-20_, but not with OVA_323-339_. Further analysis revealed that knockdown of Neat1 strongly reduced not only the proportion of Th17 cells (Fig. 3C), but also the expression of pathogenic Th17-related genes *Il17*, *Csf2*, *Il23r*, *Il1r1, Rorc*, and *Irf4*, but not *Il22* (Fig. 3D). These findings suggest that Neat1 positively affect the development of antigen-specific Th17 cells.

### Silencing of Neat1 in T cells impaired the ability of Th17 cells to induce EAU

**Fig. 4:**
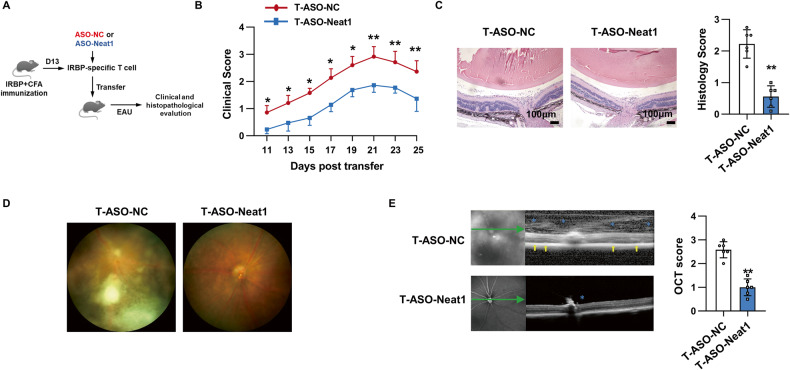
Silencing of Neat1 in Th17 cells ameliorates EAU. **A** Schematic diagram of EAU induced by adoptive transfer. **B** Clinical scores of EAU in naïve C57BL/6 mice adoptively transferred with ASO-NC (T-ASO-NC) or ASO-Neat1 (T-ASO-Neat1)-transfected Th17 cells (*n* = 6 per group). **C** Histopathological analysis of eyes from the transferred EAU mice (*n* = 6 per group). Scale bars, 100 μm. **D** Representative images of eyes in the transferred EAU mice by retinal imaging microscope. **E** Representative images showing fundus condition by SD-OCT. Hyper-reflective foci (blue asterisk) in the vitreous cavity and retinal folds (yellow arrows) were observed around the optical nerve. Data are representative of the analysis of three independent experiments. Error bars represent the mean ± SD. *p < 0.05, **p < 0.01.

To further confirm the T cell-intrinsic regulatory role of Neat1 in the pathogenesis of EAU, CD4^+^ T cells sorted from EAU mice were transfected with ASO-NC or ASO-Neat1, washed, and then co-cultured with APCs and IRBP_1-20_ under Th17-polarizing conditions. Two days later, the activated Th17 cells were adoptively transferred into naïve recipient mice which were monitored for the development of EAU (Fig. 4A). Compared with the control group, mice receiving Neat1-silenced Th17 cells displayed remarkedly decreased EAU clinical scores (Fig. 4B) and histopathology scores (0.54 ± 0.37 vs. 2.23 ± 0.45, Fig. 4C). Consistently, the fundus images and SD-OCT images taken on day 21 after transfer also confirmed alleviated EAU signs in mice that received ASO-Neat1-Th17 cells (Fig. 4D, E). Thus, Neat1 knockdown in CD4^+^ T cell alone leads to a significantly attenuated EAU, confirming the T cell-intrinsic role of Neat1 in pathogenic Th17 cell function.

### Neat1 inhibits the transcriptional activity of NonO to increase *Il17* and *Il23r* transcription

**Fig. 5:**
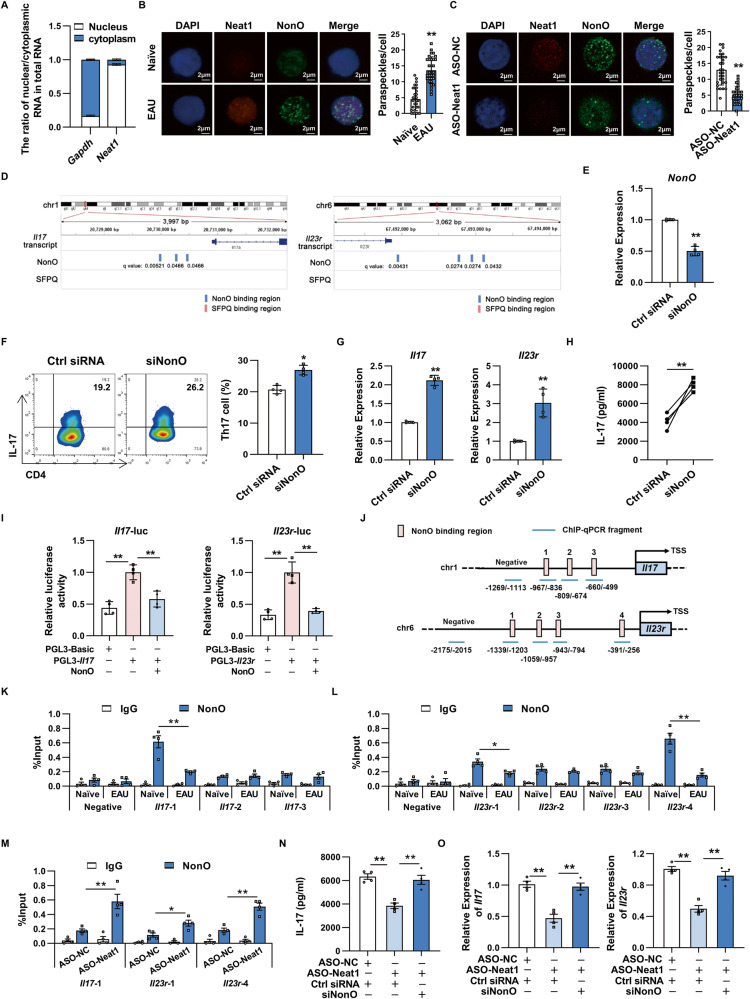
NonO is involved in Neat1 mediated antigen-specific Th17 cell responses. **A** Cytoplasmic and nuclear levels of Neat1 in CD4^+^ T cells from EAU mice (*n* = 3 per group). **B** Representative images of paraspeckles in naïve and EAU CD4^+^ T cells under confocal microscopy (*n* = 30 cells in each group). Red, Cy3-labeled Neat1 probes; green, Alexa-488-labeled secondary antibody for NonO protein. Scale bars, 2 μm. **C** Representative confocal images of paraspeckles in EAU CD4^+^ T cells upon Neat1 knockdown (*n* = 30 cells in each group). **D** Prediction of NonO and SFPQ binding sites in the *Il17* and *Il23r* promoter regions by MEME analysis. **E**–**H**, **N–O** CD4^+^ T cells isolated from immunized mice were transfected with indicated oligonucleotides and stimulated with IRBP_1-20_ in the presence of irradiated APCs under Th17-polarizing conditions. **E** Real-time qRT-PCR analysis of NonO expression (*n* = 4 per group). **F** Flow cytometric analysis of the percentages of Th17 cells (*n* = 4 per group). **G**, **O** Real-time qRT-PCR analysis of *Il17* and *Il23r* expression (*n* = 4 per group). **H**, **N** ELISA analysis of IL-17 secretion in the culture supernatants (*n* = 4 per group). **I** Luciferase activity analysis of reporter containing *Il17* or *Il23r* promoter transfected into HEK293T with the indicated plasmids (*n* = 4 per group). **J**–**M** ChIP analysis of NonO occupancy at the promoter of the *Il17* or *Il23r* gene was performed in CD4^+^ T cells (*n* = 3 per group). Blue lines 1 to 4 indicate regions detected by ChIP-qPCR. Regions without NonO binding sites (no binding site) were used as negative control. Data present at least three independent experiments. *p < 0.05, **p < 0.01.

To uncover the molecular mechanism underlying the action of Neat1 on pathogenic Th17 cell responses, we next performed cellular fractionation assays and found that Neat1 was distributed in both nuclear fractions and cytoplasmic fractions in CD4^+^ T cells from EAU mice, but mainly in the nucleus (Fig. 5A). Given that Neat1 is an essential structural component of nuclear paraspeckles [28], we next tested the existence of Neat1 in the form of a paraspeckle in the nucleus. We observed Neat1 foci in EAU CD4^+^ T cells using RNA probes that recognize Neat1. Immunofluorescence with anti-NonO antibody followed by staining of Neat1 with fluorescence in situ hybridization (FISH) demonstrated that NonO overlapped well with the Neat1 puncta, which further indicated the existence of paraspeckles in EAU CD4^+^ T cells (Fig. 5B). The number of paraspeckles was significantly increased in EAU CD4^+^ T cells compared to naïve CD4^+^ T cells (Fig. 5B). Further analysis revealed that the number of paraspeckles in EAU CD4^+^ T cells was dramatically decreased when Neat1 was silenced using antisense oligonucleotides targeting Neat1 (Fig. 5C). A recent study reported that Neat1 could promote *Il8* transcription by relocating paraspeckle protein SFPQ from the *Il8* promoter region to the paraspeckles [17]. Thus, we assumed that certain paraspeckle proteins such as SFPQ or NonO may participate in Neat1-mediated Th17 cell responses. To test this, we first performed bioinformatics analysis and found that Neat1-regulated *Il17* and *Il23r* locus contained putative NonO, but not SFPQ, binding sites (Fig. 5D), indicating that expression of *Il17* and *Il23r* may be directly regulated by NonO. As expected, we found that the mRNA expression of *Il17* and *Il23r* and the production of IL-17 were significantly increased in NonO-silencing Th17 cells (Fig. 5E–H). To further confirm this, we constructed luciferase reporters containing the *Il17* or *Il23r* promoter region. As shown in Fig. 5I, NonO overexpression dramatically decreased the promoter activities of both *Il17* and *Il23r* genes. We then employed a chromatin immunoprecipitation (ChIP) experiment to examine whether NonO binds directly and specifically to the *Il17* and *Il23r* promoters in CD4^+^ T cells from naïve or EAU mice. As presented in Fig. 5J, K, we found that NonO bound to the promoter region of *Il17* gene, more strongly, to the region 1 (−967bp/−836bp) at *Il17* gene in naïve CD4^+^ T cells, while EAU CD4^+^ T cells displayed reduced NonO binding to the region 1 at *Il17* gene. In addition, we also observed that compared with naïve CD4^+^ T cells, EAU CD4^+^ T cells exhibited reduced NonO binding to the region 1 (−1339bp/−1203bp) and region 4 (−391bp/−256bp) at *Il23r* gene (Fig. 5J, L). To confirm the interference of NonO binding activity by Neat1 further, we next examined NonO binding to *Il17* and *Il23r* promoter regions in the Neat1-silencing CD4^+^ T cells. As shown in Fig. 5M, the binding of NonO to region 1 at *Il17* gene and region 1/4 at *Il23r* gene was significantly increased in EAU CD4^+^ T cells upon Neat1 knockdown. Further analysis revealed that inhibition of NonO partly abolished the decrease of IL-17 production, and *Il17* and *Il23r* mRNA expression induced by the knockdown of Neat1 (Fig. 5N, O). Together, these data suggest that Neat1-mediated enhanced pathogenic Th17 cell responses can be regulated by modulating the transcription activity of NonO.

### Neat1 upregulates *Il17* and *Il23r* expression by regulating miR-128-3p/NFAT5 axis

**Fig. 6:**
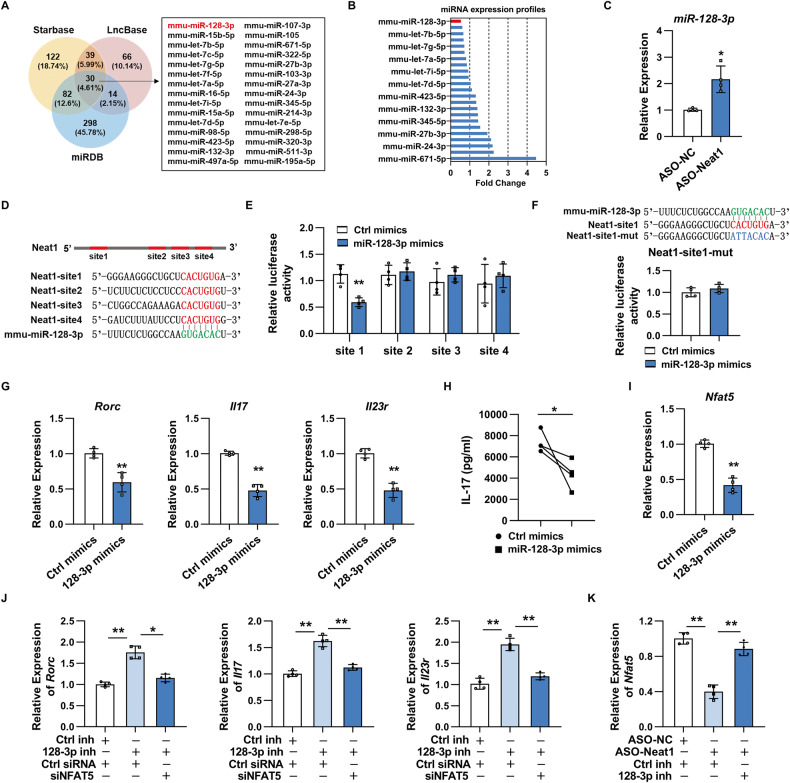
Neat1 promotes antigen-specific Th17 cell responses by regulating miR-128-3p/NFAT5 axis. **A** Venn diagram analysis of putative Neat1 target miRNAs using Starbase v3.0, DIANA-LncBase, and miRDB databases. **B** Only 23 candidate miRNAs were expressed in CD4^+^ T cells isolated from EAU mice. Microarray-based bar graphs showing the expression level of candidate miRNA in CD4^+^ T cells isolated from EAU mice relative to those from naïve mice. **C** Real-time qRT-PCR analysis of miR-128-3p expression in Th17 cells transfected with ASO-NC or ASO-Neat1 (*n* = 4 per group). **D** Sequence alignment between miR-128-3p and its potential binding sites (in red letters) in the lncRNA Neat1. **E**, **F** Luciferase activity analysis of reporter carrying Neat1 binding sites or mutant site (Mut) co-transfected into HEK293T with Ctrl mimics or miR-128-3p mimics (*n* = 4 per group). **G**–**K** CD4^+^ T cells isolated from immunized mice were transfected with indicated oligonucleotides and stimulated with IRBP_1-20_ in the presence of irradiated APCs under Th17-polarizing conditions. **G**, **J** Real-time qRT-PCR analysis of *Rorc*, *Il17* and *Il23r* expression (*n* = 4 per group). **H** ELISA analysis of IL-17 secretion in the culture supernatants (*n* = 4 per group). **I**, **K** Real-time qRT-PCR analysis of *Nfat5* expression (*n* = 4 per group). Data are presented as mean ± SD of at least three independent experiments. *p < 0.05, **p < 0.01. See also Fig. S3.

Neat1 can exert its role via sponging miRNAs to reduce miRNAs regulation on their target mRNA [29]. We next asked whether Neat1 regulated pathogenic Th17 cell responses via this mechanism. By overlapping the miRNA target prediction results from Starbase v3.0, DIANA-LncBase, and miRDB, 30 candidate miRNAs were predicted to be targets of Neat1 (Fig. 6A). Combined with the miRNA microarray data of EAU CD4^+^ T cells shown in our previous study [12], miR-128-3p was found to be the most downregulated in EAU CD4^+^ T cells (Fig. 6B). Further analysis revealed that miR-128-3p was significantly upregulated in Neat1-silenced Th17 cells (Fig. 6C), and miR-128-3p could directly bind to Neat1 via binding site 1 in luciferase reporter gene assay (Fig. 6D–F).

We then sought to determine whether miR-128-3p is functionally important for Neat1-mediated Th17 cell responses. As shown in Fig. 6G, H, miR-128-3p overexpression resulted in a significant decrease in *Rorc*, *Il17* and *Il23r* expression and IL-17 production. NFAT5, a target of miR-128-3p, is a potent inducer of pathogenic Th17 cells by activating *Rorc* transcription [30–32]. As presented in Fig. 6I, *Nfat5* expression level was remarkably decreased in EAU Th17 cells transfected with miR-128-3p mimics. Notably, miR-128-3p inhibitor mediated increased expression of *Rorc*, *Il17*, and *Il23r* was partially canceled by silencing NFAT5 (Fig. 6J), and inhibiting miR-128-3p activity partially counteracted reduced *Nfat5* expression induced by ASO-Neat1 in EAU Th17 cells (Fig. 6K). These results suggest that Neat1 serves as a sponge for miR-128-3p to promote NFAT5 expression, thus facilitating pathogenic Th17 cell responses.

## Discussion

Here, we reported that Neat1, which was upregulated significantly in CD4^+^ T cells, was an important regulator of pathogenic Th17 cell responses that contributed to the development of autoimmunity. Using the IRBP-induced model and adoptive transfer model of EAU, we further verified that both global and T cell-specific silencing of Neat1 caused alleviated EAU disease. Mechanistically, Neat1 promoted the transcription of *Il17* and *Il23r* through the sequestration of transcriptional regulator NonO in paraspeckle. In addition, it also up-regulated the expression of NFAT5 via sponging miR-128-3p, thereby promoting pathogenic Th17 cell program.

**Fig. 7:**
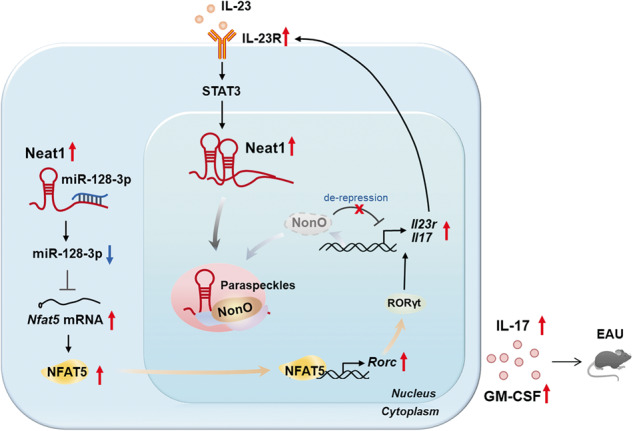
Schematic showing the molecular mechanisms by which Neat1 regulates antigen-specific Th17 cell responses in EAU. The increased expression of Neat1 is induced by IL-23 signaling, and Neat1 in turn augments IL-23R expression by regulating NonO and miR-128-3p/NFAT5, thus forming a positive feedback loop to sustain antigen-specific Th17 cell responses that contribute to the progression of EAU.

Pathogenic Th17 cells, with higher expression of *Il17*, *Csf2*, *Il23r*, and *Il1r1*, play a pivotal role in the pathogenesis of autoimmune disorders [6, 7, 33]. However, the roles of lncRNAs in the regulation of antigen-specific Th17 responses remain poorly understood. Recently, Neat1-silenced naïve T cells were found to display an impaired ability to differentiate toward Th17 cells [20, 34]. Nevertheless, the function and mechanism of Neat1 in antigen-specific Th17 cell responses have not been fully investigated. Here, we found that Neat1 expression was significantly higher in antigen-specific Th17 cells than Th1 and Th0 cells, and Neat1-silenced EAU mice displayed impaired Th17 cell responses, suggesting the possible positive role of Neat1 in antigen-specific Th17 cell responses. This was further confirmed by in vitro experiments showing that silencing Neat1 resulted in significantly reduced IL-17 production when T cells were restimulated with immunizing antigen IRBP_1-20_, but not with irrelevant antigen OVA_323-339._ These findings demonstrated that Neat1 positively regulated pathogenic Th17 cell responses. Using the adoptive transfer model of EAU, we further demonstrated that silencing of Neat1 in T cells alone restrained the pathogenic capacity of antigen-specific Th17 cells to induce EAU, highlighting the pivotal role of T cell-intrinsic Neat1 in pathogenic Th17 cell function and autoimmunity. Notably, we observed that IL-23 could enhance the expression of Neat1 via STAT3 signaling, and Neat1 in turn augmented IL-23R expression, thus forming a positive feedback loop to sustain pathogenic Th17 cell responses (Fig. 7). Given that antigen-specific Th17 cells contribute to the pathogenesis of AU, targeting Neat1 may be a potential strategy to treat AU.

Pro-inflammatory cytokines such as IL-6, IL-1β, and IL-23 produced by dendritic cells (DCs) play a crucial role in instructing pathogenic Th17 cell programs [35]. IL-6 and IL-1β are essential for priming Th17 cell development, and IL-23 confers pathogenic features to Th17 cells [7, 36]. However, limited information is available about the role of DC-intrinsic Neat1 in antigen-specific Th17 cells. Here, we observed a significant decrease in the frequency of CD40^+^CD11c^+^ cells and the expression of *Il6*, *Il1b*, and *Il23* in the spleen of Neat1-silenced EAU mice (Fig. S2A, B), suggesting that knockdown of Neat1 may form a cytokine milieu that suppresses the generation of pathogenic Th17 cells. Indeed, using DC-T co-culture experiments, we found that silencing of Neat1 in DCs significantly suppressed the expression of *Il6*, *Il1b*, and *Il23* (Fig. S2C), and Neat1-silenced DCs markedly inhibited the secretion of IL-17 and the expression of *Il17*, *Rorc*, *Il23r*, and *Csf2* in antigen-specific Th17 cells (Fig. S2D, E).

RNA-RBP interactions are involved in various biological processes via regulating gene expression [37]. Especially, the location and activity of RBPs are found to be widely regulated by non-coding RNAs including lncRNAs, which play an important role in the generation and function of immune cells [38]. For example, lncRNA-GM could bind to transcription factor Foxo1, which is important for regulating the Th17/Treg cell balance [15]. LincRNA-MAF-4 negatively regulated MAF transcription by binding to EZH2 and LSD1, thus inhibiting T cell differentiation toward Th2 [27]. Here, we found that there are RBP NonO binding sites at Neat1-regulated *Il17* or *Il23r* gene. Using combination of ChIP experiments and reporter gene assays, we further verified that NonO silenced *Il17* and *Il23r* transcription by binding directly and specifically to the promoter regions of *Il17* and *Il23r* genes, suggesting that NonO is a transcriptional repressor of *Il17* and *Il23r* genes. Importantly, we further confirmed that NonO silencing almost reversed the defective *Il17* and *Il23r* expression caused by silencing Neat1 in T cells, indicating that NonO is a key molecular downstream of the action of Neat1 on Th17 cells. Neat1 could bind to RBP NonO by a conserved structural motif G-quadruplexs to form paraspeckle in HEK293T cells [39]. Here, using combined staining of Neat1 and NonO, we firstly showed the co-localization of Neat1 with NonO in paraspeckles in CD4^+^ T cells. We also found that Neat1 knockdown led to a significant decrease in the number of paraspeckles in EAU CD4^+^ T cells. Importantly, the binding of NonO to region 1 at *Il17* gene and region 1/4 at *Il23r* gene was significantly increased in EAU CD4^+^ T cells when Neat1 was knocked down. These findings suggest that Neat1 knockdown may cause relocation of NonO from paraspeckles to chromatin, thus leading to NonO-mediated transcription repression of *Il17* and *Il23r* genes and decreased pathogenic Th17 responses in Neat1 silenced mice. Neat1 has recently been found to interact with M1 domain of RBP PGK1 and stabilize PGK1, thereby facilitating glioma progression [40]. PGK1, a newly discovered Th17 regulator, could enhance the glycolysis of CD4^+^ T cells and promote Th17 cell differentiation [41]. However, whether Neat1 affects pathogenic Th17 cell responses by interacting with PGK1 or other RBPs in EAU remained to be determined.

The mechanisms underlying Neat1 function are complex [29, 42]. Neat1 has been shown to sponge miR-214-5p to reduce LTF expression, thus promoting autophagy in lung squamous cell carcinoma cells [43]. Consistent with a recent study in cancer research [44], here we found that miR-128-3p is a functional target of Neat1 in pathogenic Th17 cells. miR-128-3p has been shown to inhibit Treg cell infiltration in gastric cancer by targeting IL-16 [45]. However, the role of miR-128-3p in the regulation of pathogenic Th17 cell responses remained unknown. Here, we found that overexpression of miR-128-3p significantly inhibited the production of IL-17 and the expression of *Il17* and *Il23r* via targeting NFAT5, suggesting the negative role of miR-128-3p/NFAT5 axis in pathogenic Th17 cell responses. Importantly, we observed that miR-128-3p silencing partially reversed decreased IL-17 production, *Il17* and *Il23r* expression induced by knockdown of Neat1 in T cells (Fig. S3A, B), indicating that miR-128-3p was also involved in Neat1 mediated enhanced pathogenic Th17 cell responses. Thus, Neat1 may positively regulate pathogenic Th17 cells by simultaneously modulating NonO, miR-128-3p, or others.

In summary, we have discovered a critical role of Neat1 in promoting antigen-specific Th17 cell responses via modulating NonO and miR-128-3p/NFAT5. The role of Neat1 in driving pathogenic Th17 cell responses and autoimmunity identifies Neat1 as a promising therapeutic target in uveitis and other Th17 cell-mediated autoimmune diseases.

## Materials and methods

### Animal, reagents, and antibodies

Pathogen-free C57BL/6 (10-week-old) mice were procured from Vital River Laboratory Animal Technology (Beijing, China). All animal care and use conformed to the Association for Research in Vision and Ophthalmology (ARVO) Statement for the Use of Animals in Ophthalmic and Vision Research, and the experimental protocols were approved by the Animal Care and Use Committee of Tianjin Medical University (ethics approval number: TJYY2019110117).

The interphotoreceptor retinoid-binding protein (IRBP) peptide 1–20 (aa 1–20 of IRBP) peptide was synthesized and purified by Sangon Biotech (Shanghai, China). Complete Freund’s adjuvant (CFA) and pertussis toxin were purchased from Sigma Aldrich (St Louis MO, USA). Mycobacterium tuberculosis H37RA was obtained from BD Biosciences (San Jose, CA, USA). The recombinant murine cytokines IL-23, IL-4, and GM-CSF were obtained from R&D Systems (Minneapolis, MN, USA). PE-conjugated, FITC-conjugated, or APC-conjugated antibodies against mouse CD4, IL-17, IFN-γ, Foxp3, CD40, and CD11c were purchased from BioLegend (San Diego, CA, USA). Anti-NonO was purchased from Proteintech (Chicago, IL, USA). The WP1066 was obtained from Calbiochem (San Diego, CA, USA), and dissolved in DMSO for storage and usage.

### EAU induction

For active induction of EAU, C57BL/6 mice were injected intraperitoneally with 800 ng pertussis toxin and immunized subcutaneously at over six spots on the tail base and flank with 200 μg IRBP_1-20_ emulsified in CFA containing 0.8 mg mycobacterium tuberculosis H37RA.

For adoptive transfer, CD4^+^ T cells from IRBP_1-20_-immunized mice were transfected with anti-sense oligonucleotides against Neat1 (ASO-Neat1) or control ASOs (ASO-NC) and restimulated with 10 μg/ml IRBP_1-20_ in the presence of antigen-presenting cells (APCs) for 48 h under Th17-polarizing conditions. Then, the activated T cells were separated and injected intraperitoneally into naïve C57BL/6 mice as described previously [11, 12]. All mice were age and weight-matched and then randomized into the different groups. There were no animal exclusion criteria.

### EAU evaluation

Clinical signs of EAU were evaluated by indirect fundoscopy three times a week and funduscopic grading of disease was performed using the scoring systems as described previously [4]. The fundus imaging evaluation was conducted with the retinal imaging system (Micron IV, Phoenix Research Labs, Pleasanton, CA, USA) and spectral-domain optical coherence tomography (SD-OCT, Heidelberg Engineering, Heidelberg, Germany). For histopathological evaluation, the whole eyes were fixed, embedded in paraffin, and sectioned along the papillary-optic nerve axis. The sections were stained with hematoxylin and eosin (H&E) and the severity of EAU was scored using previously reported criteria [4] based on the cellular infiltration and structural changes. Clinical and histopathological scoring were performed blinded.

### CD4^+^ T cell isolation, transfection, and polarization

CD4^+^ T cells isolated from the spleen and draining lymph nodes of IRBP_1-20_-immunized mice were purified by positive selection using auto-MACS separator (Miltenyi Biotec, Bergisch Gladbach, Germany) according to the manufacturer’s protocol. Then, the siRNAs (200 nM) or ASOs (300 nM) were transfected into EAU CD4^+^ T cells using Lipofectamine 2000 reagent (Thermo Fisher Scientific, Waltham, MA, USA). After transfection for 24 h, CD4^+^ T cells (1 × 10^6^ cells/well) were co-cultured with 1 × 10^6^ irradiated (30 Gy) syngeneic splenocytes as APCs, which were pre-incubated with 10 μg/ml IRBP_1-20_ for 20 min in a 24-well plate, under Th17 cell polarization (culture medium supplemented with 10 ng/ml IL-23). siRNAs, ASOs, miR-128-3p mimics, inhibitors, and corresponding negative control were designed and synthesized by Gene Pharma (Suzhou, China). All sequences used are provided in Table S1.

### Plasmid construction and lentivirus packaging

The short-hairpin RNA (shRNA) sequences that specifically targeted Neat1 were synthesized by Sangon Biotech (Shanghai, China) and inserted into lentiviral vector pLL3.7 (Addgene) at the *Hpa I* and *Xho I* sites. *NonO* coding region was inserted into the pCDH-CMV-MCS-EF1-copGFP (System Biosciences) between the *EcoR I* and *Not I* sites. All primers used are listed in Table S2.

For lentivirus packaging and production, shRNA-Neat1 (shNeat1) was transfected into human embryonic kidney 293 T cells (HEK293T) along with psPAX2 (Addgene) and pMD2.G (Addgene) using Lipofectamine 2000 (Thermo Fisher Scientific). The culture supernatants were collected and concentrated with PEG8000 (Solarbio, Beijing, China) at 48 h and 72 h post-transfection. To explore the effect of Neat1 knockdown on EAU, LV-Ctrl or LV-shNeat1 (approximately 5 × 10^7^ TU) were administered to randomly selected mice via tail vein 7 days before immunization with IRBP_1-20_.

### Flow cytometric analysis

For surface staining, cells were harvested, washed, and stained with the PE- or FITC-conjugated anti-mouse CD4 antibodies in the presence of Fc blocker (BioLegend). For intracellular staining, cells were stimulated with phorbol myristic acetate (50 ng/ml, Sigma Aldrich), ionomycin (1 μg/ml, Sigma Aldrich), and brefeldin A (1 μg/ml, Sigma Aldrich) for 4–6 h in complete RPMI 1640 medium. Then, cells were fixed and permeabilized overnight using Fixation and Permeabilization kit (eBioscience, SanDiego, CA, USA), followed by incubating with FITC-conjugated anti-mouse IL-17 antibody, PE-conjugated anti-mouse Foxp3 antibody, and APC-conjugated anti-mouse IFN-γ antibody. The stained cells were detected by flow cytometry (FACS Calibur, BD Biosciences, San Jose, CA, USA), and the data were analyzed by FlowJo software (Tree Star, Ashland, OR, USA).

### Enzyme-linked immunosorbent assay (ELISA)

The cytokines in the culture supernatants were detected with Mouse IL-17 DuoSet ELISA Kit (R&D Systems) according to the manufacturer’s instructions. Briefly, 96-well plates were coated with the capture antibodies overnight at room temperature. After washing and blocking, the diluted supernatants and recombinant cytokine standards were added to the plates and incubated for 2 h at room temperature. Then, the plates were incubated sequentially with the detection antibodies and streptavidin-HRP, as well as Substrate Reagent (R&D Systems) and Stop Solution. The absorbance was measured at 450 nm with wavelength correction set to 540 nm.

### Real-time quantitative RT-PCR (real-time qRT-PCR)

Total RNA was extracted from EAU CD4^+^ T cells using TRIzol reagent (Thermo Fisher Scientific) and then cDNA was synthesized with RevertAid First Strand cDNA Synthesis Kit (Thermo Fisher Scientific) according to the manufacturer’s protocol. Real-time qRT-PCR was performed using SYBR Green Master Mix and LightCycler® 480 II real-time PCR instrument (Roche, Basel, Switzerland). The stem-loop primer method was used for the quantification of miR-128-3p and its relative expression was normalized to U6 snRNA levels within each sample. The primer sequences were as follows: miR-128-3p RT primer: 5′-GTCGTATCCAGTGCAGGGTCCGAGGTATTCGCACTGGATACGACAAAGAG-3′; miR-128-3p Forward primer: 5′-CGCGTCACAGTGAACCGGT-3′; miR-128-3p Reverse primer: 5′-AGTGCAGGGTCCGAGGTATT-3′. For mRNA analysis, *Gapdh* was used as an internal control, and the relative expression levels of each sample were calculated using the 2^–ΔΔCt^ method. The gene-specific primers for real-time qRT-PCR are listed in Table S3.

### Cellular fractionation assay

Cellular fractionation assay was performed using NE-PER Nuclear and Cytoplasmic Extraction Reagents (Thermo Fisher Scientific) according to the manufacturer’s protocol. Briefly, CD4^+^ T cells were incubated on ice for 15 min with ice-cold cytoplasmic extraction reagent I (CER I), and then added CER II in the samples. After incubating on ice for 1 min, the lysate was centrifuged at 16,000 × *g* for 5 min. The supernatant was collected as a cytoplasmic fraction. Next, the pellet was resuspended in the nuclear extraction reagent and centrifuged at 12,000 × *g* for 5 min. The supernatant was collected as the nuclear fraction. RNA from all fractions was isolated using the TRIzol reagent as described above.

### Chromatin immunoprecipitation

Chromatin immunoprecipitation (ChIP) was performed using the chromatin immunoprecipitation assay kit (Abcam, MA, USA). Briefly, CD4^+^ T cells were crosslinked with 1% formaldehyde for 10 min at room temperature and then suspended in Cell Lysis Buffer (Abcam). After sonication with 10 s pulses 30 times on ice, 10% of chromatin used for each ChIP reaction was kept as input DNA, and the remaining was incubated with ChIP buffer containing 5 μg antibodies against NonO (Proteintech, Chicago, IL, USA) or control IgG overnight at 4 °C. The samples were mixed with Protein A agarose beads for 1 h at 4 °C on a rotating platform. Then, the DNA fragment bound with the above antibodies was purified using DNA purifying slurry and subjected to ChIP-qPCR analysis addressing the *Il17* and *Il23r* promoter regions. ChIP-qPCR primers are listed in Table S4.

### Luciferase assay

The mouse *Il17* or *Il23r* promoter was cloned into pGL3-Basic (Promega, Madison, WI, USA) between the *Kpn I* and *Xho I* site. The Neat1 fragment containing the putative miR-128-3p binding site or mutant (mut) Neat1 fragment was amplified and cloned into pMIR-Report (Promega) between the *Hind III* and *Spe I* site, respectively. For the dual luciferase reporter assay, HEK293T cells (2.5 × 10^4^ cells/well) were seeded in 96-well plates, and co-transfected with indicated plasmids and oligonucleotides using Lipofectamine 2000 (Thermo Fisher Scientific), and pRL-TK vector (Promega) was used as the internal control. Forty-eight hours after transfection, luciferase activity was measured with the Dual-Luciferase Reporter Assay System (Promega) according to the manufacturer’s protocol.

### Immunofluorescence (IF) combined with Fluorescence in situ hybridization (FISH)

IF combined with FISH was performed in an RNase-free environment as previously described [46, 47], and RNase-free PBS was used at all times. For IF, CD4^+^ T cells were fixed with 4% paraformaldehyde (Solarbio) for 15 min at room temperature and then permeabilized with 0.3% TritonX-100 (Solarbio) for 10 min. After blocking with 5% goat serum (Solarbio), CD4^+^ T cells were incubated with rabbit anti-NonO primary antibody (Proteintech) overnight at 4 °C. Then, the cells were incubated with the goat anti-rabbit Alexa Fluor® 488 (Abcam). After IF completion, cells were hybridized with Cy3-labeled Neat1 probe mix (Gene Pharma) overnight at 37 °C. Cells were washed in wash buffer followed by 2 × SSC and 1 × SSC to remove unhybridized probes, and the nuclei were counterstained with DAPI (Sigma Aldrich). Fluorescence images were obtained using a confocal fluorescence microscope (LSM800, Zeiss, Oberkochen, Germany) with a ×63 oil immersion objective.

### Bioinformatics analysis

GSE198533 (submission date: March 13, 2022; last update date: June 25, 2022) and GSE17114 (submission date: July 15, 2009; last update date: March 25, 2019) were downloaded from Gene Expression Omnibus (GEO) database (http://www.ncbi.nlm.nih.gov/geo/). The GSE198533 was generated on the GPL24676 platform, including peripheral blood mononuclear cells (PBMCs) from 9 Behçet’s disease (BD) patients and 10 healthy controls. The GSE17114 was generated on the GPL570 platform, containing PBMCs from 15 BD patients and 14 healthy controls. Differentially expressed lncRNAs in PBMCs between AU patients and controls were identified by analyzing raw data of GSE198533 and GSE17114 using limma package from R/Bioconductor software with a Benjamini-Hochberg correction [48]. Fold change >1.5 and adj. *P* < 0.05 were set as the cutoff criteria for screening upregulated lncRNAs. The NonO or SFPQ binding sites of the *Il17* and *Il23r* promoters were predicted by the FIMO tool on MEME Suite (https://meme-suite.org/meme/tools/fimo) [49].

miRNA targets of Neat1 were predicted using bioinformatics websites, including DIANA-LncBase (www.microrna.gr/LncBase) [50], miRDB (http://www.mirdb.org/) [51], and Starbase v3.0 (http://Starbase.sysu.edu.cn/index.php) databases [52].

### Generation of dendritic cells

Bone marrow-derived dendritic cells (DCs) were generated by in vitro differentiation of bone marrow cells as previously described [53]. Briefly, bone marrow cells were flushed from femurs and tibias of mice and cultured in 12-well plate (2 × 10^6^ cells per well) with complete RPMI 1640 medium supplemented with 10 ng/ml granulocyte-macrophage colony-stimulating factor (GM-CSF; R&D system, Minneapolis, MN, USA) and 10 ng/ml interleukin-4 (IL-4; R&D system). On day 6, non-adherent cells were harvested for phenotyping and further experiments.

For DCs transfection, DCs were transfected with ASO-NC or ASO-Neat1 in 24-well plate (2.5 × 10^5^ cells per well) using Lipofectamine 2000 (Thermo Fisher Scientific). At 24 h after transfection, DCs were treated with 100 ng/mL of lipopolysaccharide (LPS) and subjected to real-time qRT-PCR analysis.

For co-cultured study, the transfected DCs were pre-incubated with IRBP_1-20_ (10 μg/ml) for 20 min, and then co-cultured with CD4^+^ T cells at a ratio of 1:10 under Th17 cell polarization for 2 days or 8 days. Then the cells were collected for real-time qRT-PCR or flow cytometry analysis.

### Statistical analysis

SPSS version 26.0 software (IBM Corporation, Somers, NY, USA) was used for statistical analysis. All experiments were repeated 3 times or more, and the data were shown as mean ± standard deviation (SD). Student’s *t*-test, One-way or Two-way ANOVA, Kruskal–Wallis test, and Mann–Whitney *U* test were applied accordingly based on the number of groups and the normality of the data. Only *p* < 0.05 was considered to be statistically significant.

## Supplementary information

Supplementary Information

## Data Availability

We downloaded the microarray profiles (GSE198533 and GSE17114) from the GEO database. We used the FIMO tool on MEME Suite to predict the transcriptional factor binding sites in *Il17* and *Il23r* promoter regions. All the other data supporting the findings of this study are available within the article and its supplementary information files or available from the corresponding author upon reasonable request.
