# Supplementary material for: LncRNA Neat1 targets NonO and miR-128-3p to promote antigen-specific Th17 cell responses and autoimmune inflammation
Source: Cell Death Dis. 2023 Sep 16;14(9):610. doi: 10.1038/s41419-023-06132-0 (PMC10505237; doi:10.1038/s41419-023-06132-0)
Supplement: Supplementary file 1 — Supplementary Information [file 41419_2023_6132_MOESM1_ESM.pdf]

## Supplementary Information

# LncRNA Neat1 targets NonO and miR-128-3p to promote antigen-specific Th17 cell responses and autoimmune inflammation

Figure S1. Silencing Neat1 promotes Treg cells *in vivo*.

Figure S2. Silencing Neat1 suppressed dendritic cells-driven Th17 cell responses.

Figure S3. miR-128-3p is involved in Neat1 mediated pathogenic Th17 cell responses.

Table S1. List of oligonucleotides used in this study.

Table S2. List of primer sequences for plasmid construction.

Table S3. Sequences of primers for real-time qRT-PCR.

Table S4. List of primer sequences for ChIP-qPCR.

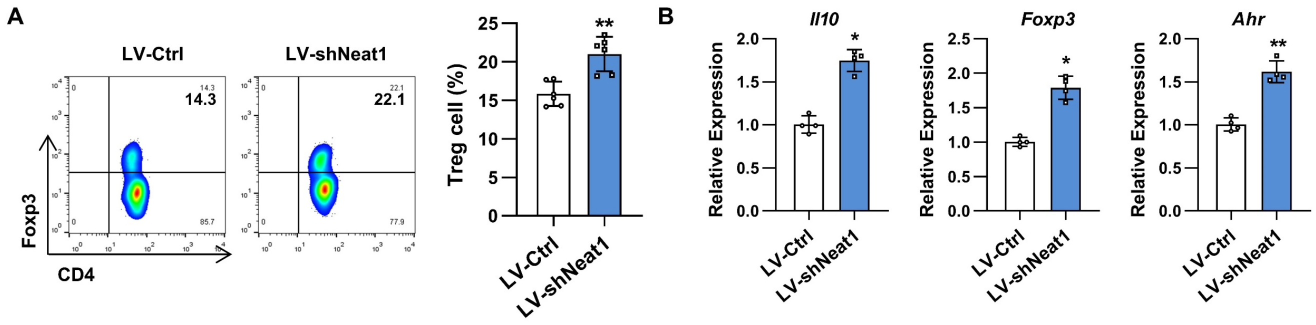


**Figure S1. Silencing Neat1 promotes Treg cells *in vivo*.**

(**A**) Flow cytometric analysis of the percentages of Treg cells in T cells from lentivirus-infected EAU mice (n=6 per group).

(**B**) Real-time qRT-PCR analysis of Treg signature genes in T cells from lentivirus-infected EAU mice (n=4 per group).

The data shown are representative of at least three independent experiments. The bar graph shows mean ± SD. *p<0.05, **p<0.01.

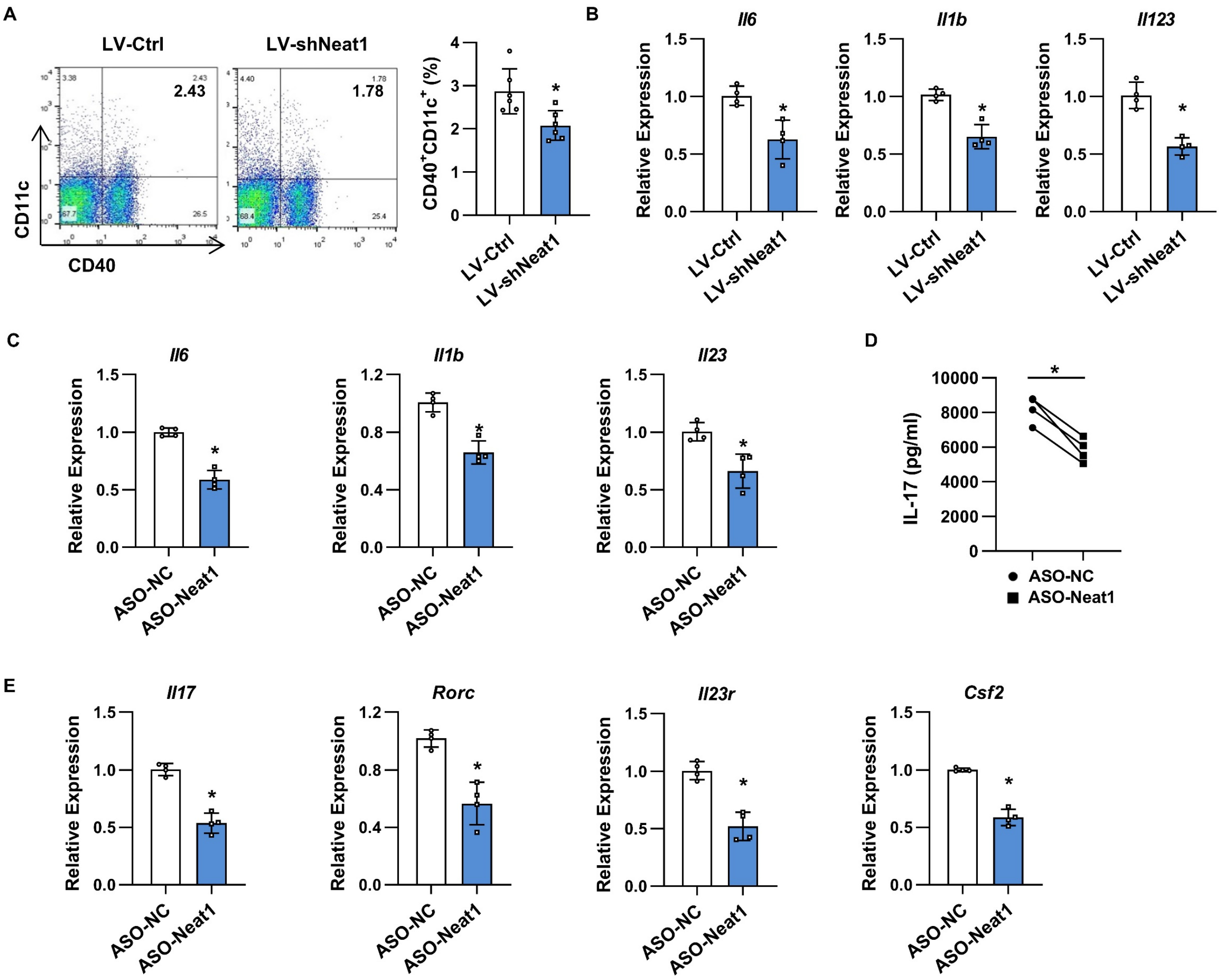


**Figure S2. Silencing Neat1 suppressed dendritic cells-driven Th17 cell responses.**

(**A**) Flow cytometric analysis of the percentages of $CD40^{+}$ $CD11c^{+}$ cells in the spleen of lentivirus-infected EAU mice (n=6 per group).

(**B**) Real-time qRT-PCR analysis of Th17 cell-polarizing cytokines expression in the spleen from lentivirus-infected EAU mice (n=4 per group).

(**C**) DCs transfected with ASO-NC or ASO-Neat1 were stimulated with 100 ng/ml LPS for 24h. Real-time qRT-PCR analysis of Th17-polarizing cytokines expression (n=4 per group).

(**D-E**) DCs treated with ASO-Neat1 or ASO-NC were co-cultured with $CD4^{+}$ T cells from EAU mice under Th17-polarizing conditions. (**D**) ELISA analysis of IL-17 secretion in the culture supernatants (n=4 per group). (**E**) Real-time qRT-PCR analysis of Th17-related gene expression (n=4 per group).

The data shown are representative of at least three independent experiments. The bar graph shows mean ± SD. *$p<0.05$, **$p<0.01$.

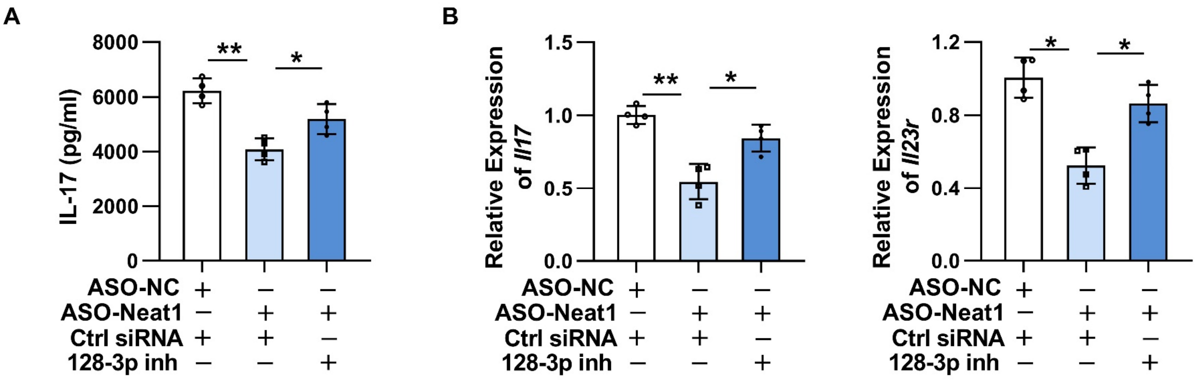


**Figure S3. miR-128-3p is involved in Neat1 mediated pathogenic Th17 cell responses.**

$CD4^+$ T cells isolated from immunized mice were transfected with indicated oligonucleotides and stimulated with $IRBP_{1-20}$ in the presence of irradiated APCs under Th17-polarizing conditions.

(**A**) ELISA analysis of IL-17 secretion in the culture supernatants (n=4 per group).

(**B**) Real-time qRT-PCR analysis of *Il17* and *Il23r* expression (n=4 per group).

Data are presented as mean ± SD of at least three independent experiments. *p<0.05, **p<0.01.

**Table S1. List of oligonucleotides used in this study.**

| Name | Sense sequence (5'-3') | Antisense sequence (5'-3') |
|---|---|---|
| Ctrl siRNA | UUCUCCGAACGUGUCACGU | ACGUGACACGUUCGGAGAA |
| NonO siRNA | CCCACCAACAACUGAACGU | ACGUUCAGUUGUUGGUGGG |
| NFAT5 siRNA | GGCACAACAGCAGUUAUCG | CGAUAACUGCUGUUGUGCC |
| Ctrl mimics | UUCUUCGAACGUGUCACGU | ACGUGACACGUUCGGAGAA |
| miR-128-3p mimics | UCACAGUGAACCGGUCUCUUU | AGAGACCGGUUCACUGUGAUU |
| Ctrl inhibitor | CAGUACUUUUGUGUAGUACAA | |
| miR-128-3p inhibitor | AAAGAGACCGGUUCACUGUGA | |
| ASO-NC | CCTTCCCTGAAGGTTCCTCC | |
| ASO-Neat1 | AAGATGCAGCAGTCGA | |

**Table S2. List of primer sequences for plasmid construction.**

| Name | Forward Primer (5'–3') | Reverse Primer (5'–3') |
|---|---|---|
| PGL3-*Il17* | CGGGGTACCAAGAAGTGTCAGAAAAGCAAAC | CCCAAGCTTATACGGTTTTACTACCTCTGTGG |
| PGL3-*Il23r* | CGGGGTACCCACTCACCATTCGCCCTCAAG | CCGCTCGAGATGGGAAGTGGCATTATTAGGA |
| LV-NonO | CGGAATTCGCCACCATGCAGAGCAATAAAGCCTTTAACTTGG | ATAAGAATGCGGCCGCCTAATATCGGCGGCGTTTATTTGGAGC |
| *Neat1*-site1 | GGACTAGTAAGATCGCTCATTGGAACAG | CCCAAGCTTGTGCCATTATCCCATGACTC |
| *Neat1*-site2 | GGACTAGTGTTAGAGGACCTTGACCGAC | CCCAAGCTTGGCTCTTGGGTTCAATTTACT |
| *Neat1*-site3 | GGACTAGTAAATGAAGGTCAGCCAACAC | CCCAAGCTTTTAGCCCTAACTCAAGTCCAA |
| *Neat1*-site4 | GGACTAGTATGCTTGCCAACCTGTGAAC | CCCAAGCTTACCTGCGATGGGATGGAGAA |
| *Neat1*-site1-mut | GGACTAGTGGAAGGGCTGCTATTACACAGCTGACAATGGCTGCAGAC | CCCAAGCTTCCAGCTCCTCAGGCAGACAGAATTGC |

**Table S3. Sequences of primers for real-time qRT-PCR.**

| Gene Name | Forward Primer (5'–3') | Reverse Primer (5'–3') |
|---|---|---|
| *Gapdh* | CATGGCCTTCCGTGTTCCTA | GCGGCACGTCAGATCCA |
| *Il17* | CCTGGCGGCTACAGTGAAG | TTTGGACACGCTGAGCTTTG |
| *Rorc* | CCTCAGCGCCCTGTGTTTT | GAGAACCAGGGCCGTGTAGA |
| *Irf4* | TGCCCCTGACCAGTCACA | CCTCGTGGGCCAAACGT |
| *Csf2* | CACCCGCTCACCCATCAC | TTCTTTGATGGCCTCTACATGCT |
| *Il23r* | CAGAGGACATCCTGCTTCAGGTA | GATGGCCAAGAAGACCATTCC |
| *Il1r1* | CCTCGGAATGAGACGATCGA | CGTGACGTTGCAGATCAGTTG |
| *Il22* | CTTTCCTGACCAAACTCAGCAA | TGGTCGTCACCGCTGATG |
| *Il10* | GGACAACATACTGCTAACCGACTC | CCTGGGGCATCACTTCTACC |
| *Foxp3* | CATTGGTTTACTCGCATGTTCG | TGTGGCGGATGGCATTCTT |
| *Ahr* | AATCCCACATCCGCATGATT | TTTGCAAGAAGCCGGAAAAC |
| *Il23* | CATAGCTGCCCGGGTCTTT | GGCACTAAGGGCTCAGTCAGA |
| *Il6* | CCACGGCCTTCCCTACTTC | TTGGGAGTGGTATCCTCTGTGA |
| *Il1b* | AGTTGACGGACCCCAAAAGA | GGACAGCCCAGGTCAAAGG |
| *NonO* | TTACAGTCCGCAACCTTCCTCAG | CCACAATGACTACAGCCCTCTCC |
| *Nfat5* | TACAGCCTGAAACCCAACACC | GACCAGAATCATTGCCCACA |
| *Neat1* | TTGGGACAGTGGACGTGTGG | TCAAGTGCCAGCAGACAGCA |

**Table S4. List of primer sequences for ChIP-qPCR.**

| Name | Forward Primer (5'–3') | Reverse Primer (5'–3') |
|---|---|---|
| ChIP-*Il17-1* | CTGAAGAGCTGGGACCTAATG | GCTCTCTCATGTTCTCTCCTCTC |
| ChIP-*Il17-2* | ATCTCCTCCTGTTAGTAGTCTCCA | GGAGGAAGGAAATGAAGTGAAA |
| ChIP-*Il17-3* | TGATACCGAACCTCAAAACAGC | CAACTGTTTTTCCTTGAATGTCTAT |
| ChIP-*Il17*-negative | CAGGAAGCTAGAGAATGGGAAA | GCTGGCTGGTAACTCTGACTTT |
| ChIP-*Il23r*-1 | GTGAAGGGATGAACATGAAGCA | GAAGATTGCCAACAAATGCCA |
| ChIP-*Il23r*-2 | TTTGTTGTTCTTTCACTCACCATT | CTTAACACAGACACCGTGCTTC |
| ChIP-*Il23r*-3 | TGAGTATAGGGTTGGGAAATGAG | GTTGGGGAGAATAAGGAGTGC |
| ChIP-*Il23r*-4 | TCCAACAAGACCACACCTCCT | GCACATAGGAGACAGAGGTAAGCA |
| ChIP-*Il23r*-negative | GGGAGGAATTTCTTATTTCTTTTG | ACTCTAAGTAAGCCCCCAAGTAAT |
